# Enhancer of mRNA Decapping protein 4 (EDC4) interacts with replication protein a (RPA) and contributes to Cisplatin resistance in cervical Cancer by alleviating DNA damage

**DOI:** 10.1186/s41065-020-00154-w

**Published:** 2020-10-14

**Authors:** Xiaoling Wu, Youwen Zhong, Qing Chen, Xin Zhang, Hua Zhang

**Affiliations:** 1 Department of Obstetrics and Gynecology, The Second Affiliated Hospital of Xi’an Jiaotong University, No. 157 Xiwu Road, Xincheng District, Xi’an City, 710004 Shaanxi Province China; 2 School of Economics and Finance, Xi’an Jiaotong University, Xi’an City, 710061 Shaanxi Province China

**Keywords:** Cervical Cancer, Cisplatin resistance, Enhancer of mRNA decapping protein 4 (EDC4), Replication protein a (RPA), DNA damage

## Abstract

**Background:**

Cervical cancer (CC) is the third most common gynecological malignancy around the world. Cisplatin is an effective drug, but cisplatin resistance is a vital factor limiting the clinical usage of cisplatin. Enhancer of mRNA decapping protein 4 (EDC4) is a known regulator of mRNA decapping, which was related with genome stability and sensitivity of drugs. This research was to investigate the mechanism of EDC4 on cisplatin resistance in CC. Two human cervical cancer cell lines, HeLa and SiHa, were used to investigate the role of EDC4 on cisplatin resistance in vitro. The knockdown or overexpression of EDC4 or replication protein A (RPA) in HeLa or SiHa cells was performed by transfection. Cell viability was analyzed by MTT assay. The growth of cancer cells was evaluated by colony formation assay. DNA damage was measured by γH2AX (a sensitive DNA damage response marker) immunofluorescent staining. The binding of EDC4 and RPA was analyzed by immunoprecipitation.

**Results:**

EDC4 knockdown in cervical cancer cells (HeLa and SiHa) enhanced cisplatin sensitivity and cisplatin induced cell growth inhibition and DNA damage. EDC4 overexpression reduced DNA damage caused by cisplatin and enhanced cell growth of cervical cancer cells. EDC4 could interact with RPA and promote RPA phosphorylation. RPA knockdown reversed the inhibitory effect of EDC4 on cisplatin-induced DNA damage.

**Conclusion:**

The present results indicated that EDC4 is responsible for the cisplatin resistance partly through interacting with RPA in cervical cancer by alleviating DNA damage. This study indicated that EDC4 or RPA may be novel targets to combat chemotherapy resistance in cervical cancer.

**Graphical abstract:**

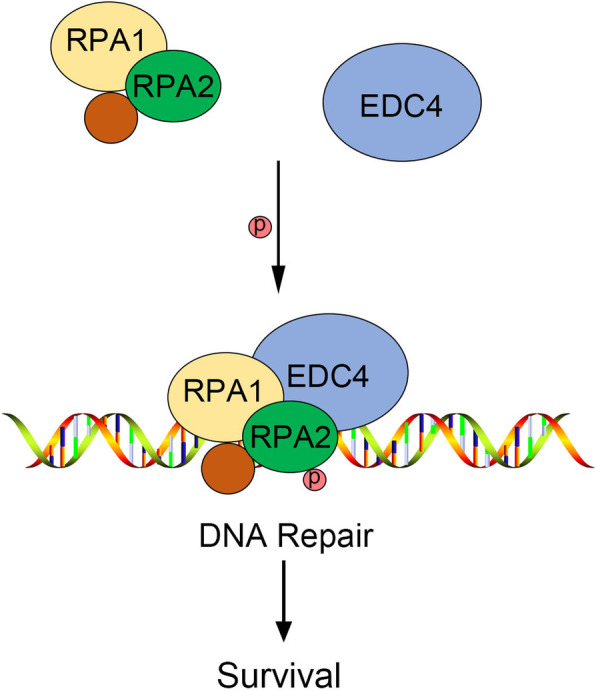

## Background

Cervical cancer (CC) is the third most common gynecological malignancy around the world. About one-third of the new cases are found in China, and showing an increasing incidence rate of young patients and early stages every year [1–3]. Although the prevention and treatment have been improved recently, but the prognosis of advanced cervical cancer is poor and prone to recurrence and metastasis [4, 5]. Cisplatin (Cis-Dichlorodiamineplatinum, CDDP) is a commonly used chemotherapeutic agent in clinic and one of the most effective drugs for treatment of advanced or recurrent CC. At the beginning of CC, patients treated with cisplatin got good effect, but many patients gradually became cisplatin-resistant [6]. High incidence of drug resistance is the most important factor limiting the clinical usage of cisplatin. Thus understanding the mechanism of cisplatin resistance in CC is of vital importance.

Enhancer of mRNA decapping protein 4 (EDC4) is a known regulator of mRNA decapping to function in the mRNA P-bodies within the cytoplasm. Hazir Rahman found that EDC4 as an interacting partner of mTORC1, which is a rapamycin sensitive complex involved in the process of energy synthesis, translation, transcription, and lipid biosynthesis [7]. EDC4 also involved in the regulation of immune system. Knocking down EDC4 or Dcp1a (components of P-bodies), reduced the production of IL-6, without decreasing the amount of IL-6 mRNA in M1-THPs. This demonstrated that EDC4 is critical in the posttranscriptional regulation of IL-6 [8]. Besides, previous research has shown that EDC4 deficiency induced the genome instability and hypersensitivity to DNA interstrand cross-linking drugs [9]. However, its detail mechanism of drug resistance is still unclear. This study focuses on the role of EDC4 in cisplatin sensitivity. Replication protein A (RPA), a eukaryotic single-stranded DNA (ss-DNA)-binding protein, binds with high affinity to ssDNA and is responsible for regulating DNA replication, homologous recombination, nucleotide excision repair and other DNA metabolism [10]. RPA-ssDNA responses to DNA damages to alleviate replication stress [11]. RPA exhaustion represents a major determinant of cisplatin sensitivity in high-grade serous ovarian cancer cells [12]. Researchers demonstrated that RPA deficiency increased the cisplatin sensitivity, and RPA overexpression led to cisplatin resistance [12]. Inhibiting the activity of RPA can prevent cell cycle progression, induce cytotoxicity, and increase the efficacy of chemotherapeutic DNA-damaging agents [13]. This research was to study the effects of EDC4 on cisplatin resistance and whether it’s related to RPA.

## Results

### EDC4 knockdown of cervical cancer cells enhanced cisplatin sensitivity

**Fig. 1:**
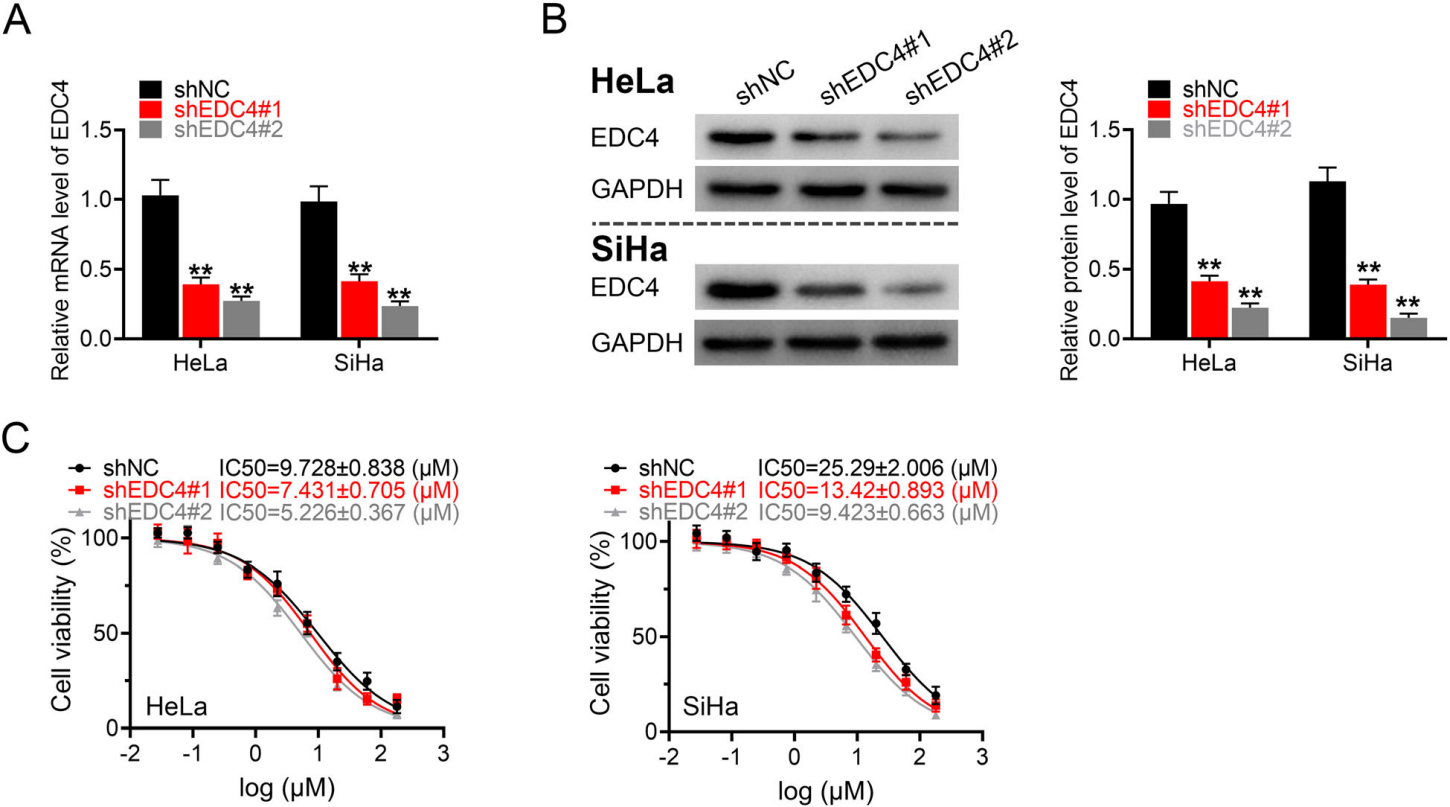
EDC4 knockdown of cervical cancer cells enhanced cisplatin sensitivity. **a** The level of EDC4 in HeLa and SiHa cells after EDC4 knockdown analyzed by RT-PCR; **b** The expression of EDC4 in HeLa and SiHa cells after EDC4 knockdown analyzed by Western blot. **c** The cell viability of EDC4 knockdown cells measured by MTT. **p < 0.01 vs. control

To investigate whether EDC4 is related with cisplatin sensitivity, we depleted EDC4 in two human cervical cancer cell lines (HeLa and SiHa) by short hairpin RNA (shRNA) of two independent shRNA sequences (shEDC4#1 and shEDC4#2). The knockdown efficacy was confirmed by RT-PCR and Western blot. As shown in Fig. 1a (gene transcription) and Fig. 1b (protein expression), the depletion levels of EDC4 of two sequences were both over 50% (*p* < 0.01 vs. control) in HeLa and SiHa cells, and sequence 2 was more potent for EDC4 knockdown. EDC4 depletion decreased the IC50 of cisplatin of HeLa (from 9.728 μM to 5.226 μM) and SiHa cells (from 25.29 μM to 9.423 μM), which indicated that the cancer cells were more sensitive to drug in terms of cell survival (Fig. 1c).

### EDC4 knockdown enhanced cisplatin induced cell growth inhibition and DNA damage

**Fig. 2:**
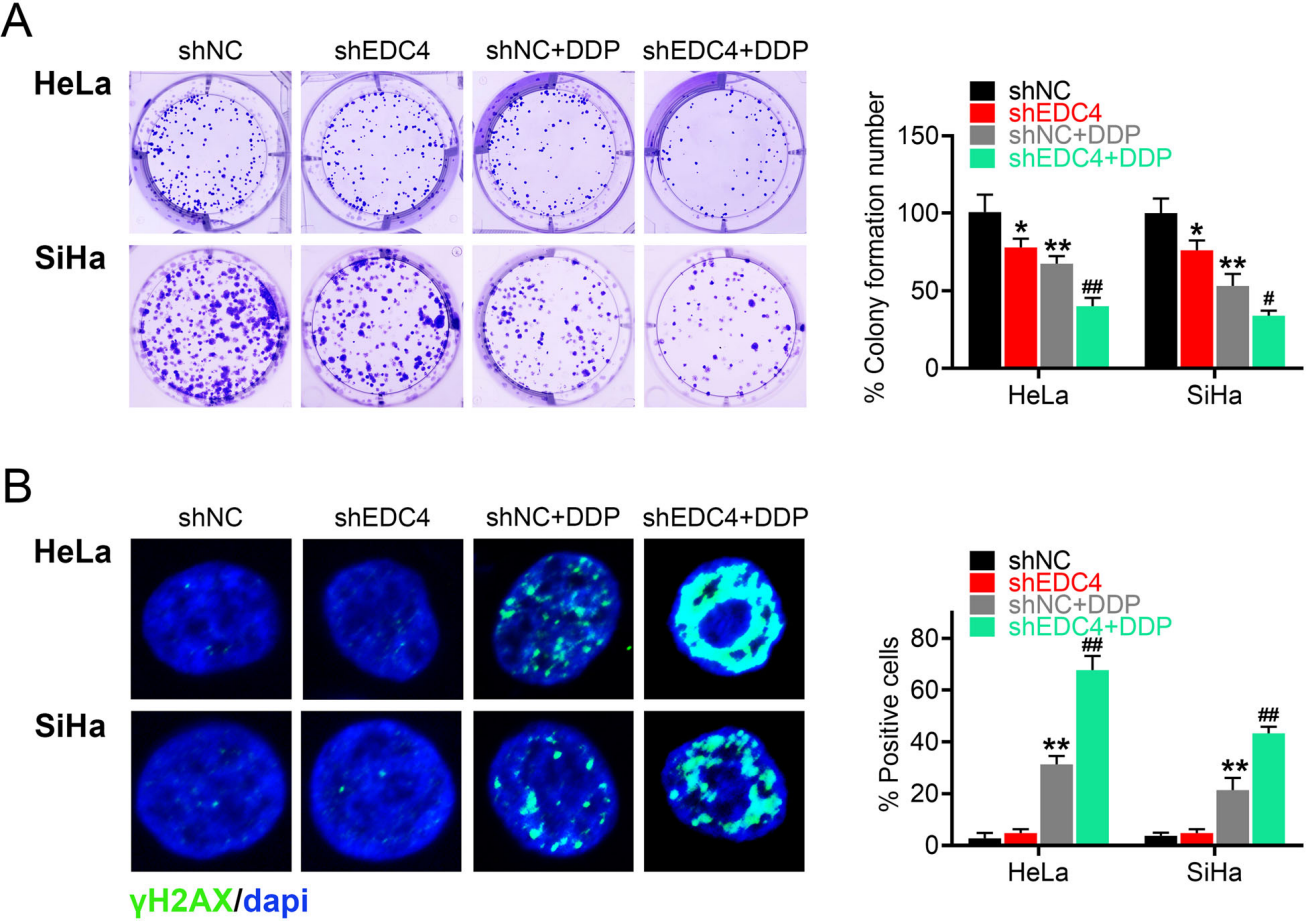
EDC4 knockdown enhanced cisplatin induced cell growth inhibition and DNA damage. **a** The cell growth of HeLa and SiHa (with EDC4 knockdown) after treated with DDP evaluated by colony formation assay. **b** The DNA damage of HeLa and SiHa (with EDC4 knockdown) analyzed by γH2AX immunofluorescent staining. *p < 0.05, **p < 0.01 vs. shNC; ^#^*p* < 0.05, ^##^*p* < 0.01 vs. shNC + DDP

To determine the mechanism of EDC4 on cisplatin resistance, the colony formation of cancer cells was first analyzed after EDC4 knockdown (shEDC4#2). Colony formation assay (Fig. 2a) showed that cisplatin (shEDC4 + DDP) significantly inhibited colony formation compared with negative control (shNC) in HeLa and SiHa cells (*p* < 0.05 for HeLa and *p* < 0.01 for SiHa), and EDC4 knockdown exhibited more potent inhibitory effect to the colony formation compared with cisplatin alone (shNC+DDP) (*p* < 0.01 for HeLa and *p* < 0.05 for SiHa). Besides, the DNA damage was measured by γH2AX (a sensitive DNA damage response marker) immunofluorescent staining. The results of γH2AX staining showed that cisplatin induced the amount of γH2AX positive cells (shNC+DDP vs. ShNC, *p* < 0.01) of HeLa and SiHa, and EDC4 knockdown obviously increased the intensity of γH2Ax (shEDC4 + DDP vs. shNC+DDP, *p* < 0.01) (Fig. 2b). These results suggested that EDC4 knockdown enhanced cisplatin induced cell growth inhibition and DNA damage.

### EDC4 overexpression reduced DNA damage caused by cisplatin and enhanced cell growth of cervical cancer cells

**Fig. 3:**
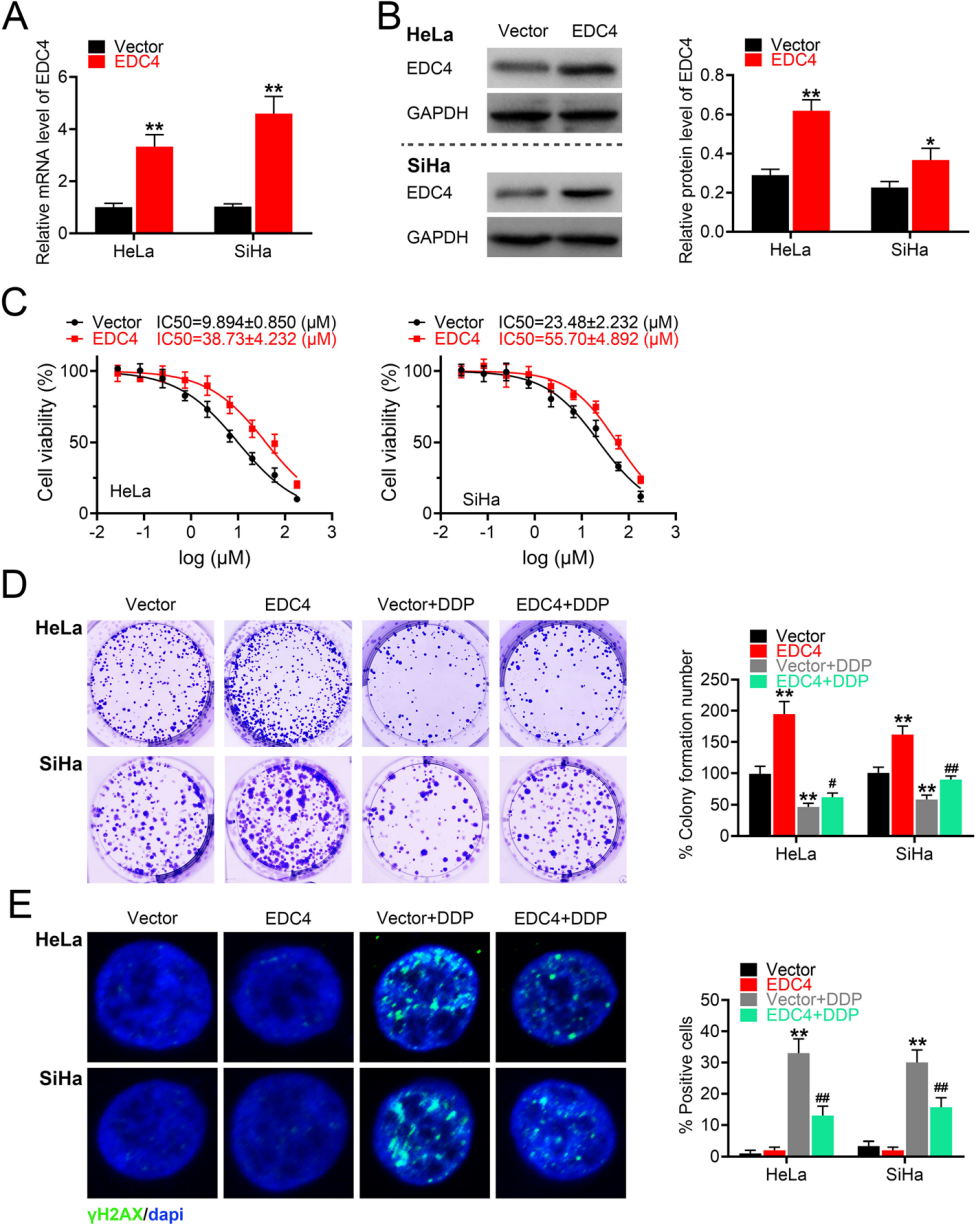
EDC4 overexpression reduced DNA damage caused by DDP and enhanced cell growth of cervical cancer cells. **a** The level of EDC4 in HeLa and SiHa cells after EDC4 overexpression analyzed by RT-PCR; **b** The expression of EDC4 in HeLa and SiHa cells after EDC4 overexpression analyzed by Western blot. **c** The cell viability of EDC4 overexpressed cells measured by MTT. **d** The cell growth of HeLa and SiHa (with EDC4 overexpression) after treated with DDP evaluated by colony formation assay. (E) The DNA damage of HeLa and SiHa (with EDC4 overexpression) analyzed by γH2AX immunofluorescent staining. *p < 0.05, **p < 0.01 vs. Vector; ^#^*p* < 0.05, ^##^*p* < 0.01 vs. Vector + DDP

To further confirm the effects of EDC4 on cisplatin sensitivity, EDC4 overexpressed cells of two human cervical cancer cell lines (HeLa and SiHa) were established. The transfection efficacy was confirmed by RT-PCR and Western blot. As shown in Fig. 3a (gene transcription) and Fig. 3b (protein expression), the level of EDC4 in HeLa and SiHa cells were both increased markedly. The IC50s of EDC4 overexpressed cells were both increased in HeLa (from 9.894 μM to 38.73 μM) and SiHa cells (from 23.48 μM to 55.70 μM) (Fig. 3c), which indicated that EDC4 overexpression induced the cisplatin resistance. Furthermore, the cell growth inhibition and DNA damage were analyzed in EDC4 overexpressed cells. As shown in Fig. 3d, EDC4 overexpressed cells exhibited more colony formation compared with negative control (EDC4 vs. Vector, *p* < 0.01). Cisplatin decreased the amount of the colony of cancer cells, but was reversed by EDC4 overexpression (Vector + DDP vs. EDC4 + DDP, *p* < 0.05 for HeLa, *p* < 0.01 for SiHa). Besides, EDC4 overexpression did not induce the DNA damage, as the amount of γH2AX positive cells had no obviously changes. Cisplatin induced the increasing of γH2AX positive cells (Vector + DDP vs. Vector, *p* < 0.01), while EDC4 overexpression inhibited the effects of cisplatin (EDC4 + DDP vs. Vector+DDP, *p* < 0.01) (Fig. 3e). These results suggested that EDC4 overexpression reversed the effects of cisplatin on cell growth.

### EDC4 interacted with RPA and promotes RPA phosphorylation

**Fig. 4:**
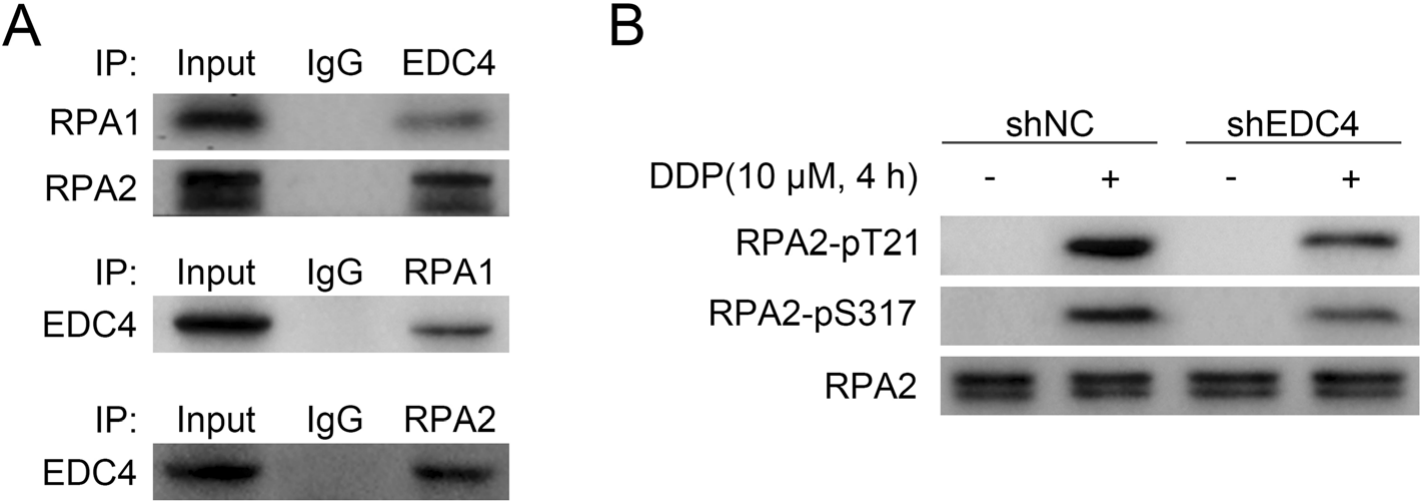
EDC4 interacted with RPA and promotes RPA phosphorylation. **a** The interaction of EDC4 and RPA1 or RPA2 was analyzed by immunoprecipitation. **b** The phosphorylation of RPA was analyzed by Western blot

As EDC4 was related with cisplatin induced DNA damage, we further study that whether it interacted with RPA, a protein binds with ssDNA and responsible for DNA metabolism. To achieve the goal, the physical EDC4-RPA interaction was confirmed by co-immunoprecipitation assay (Fig. 4a). The results showed that EDC4 could bind with RPA subunit proteins, including RPA1 and 2, and more obvious with RPA2. Further, the phosphorylation (or activation) of RPA was analyzed by Western blot (Fig. 4b). Cisplatin treatment resulted in increased phosphorylation of RPA2 (two phosphorylation site, T21 and S318), indicating a response to DNA damage, while EDC4 knockdown (shEDC4 group) decreased the phosphorylation of RPA2. These results indicated that EDC4 could interact with RPA and promote RPA phosphorylation.

### RPA knockdown reversed the inhibitory effect of EDC4 on cisplatin-induced DNA damage

**Fig. 5:**
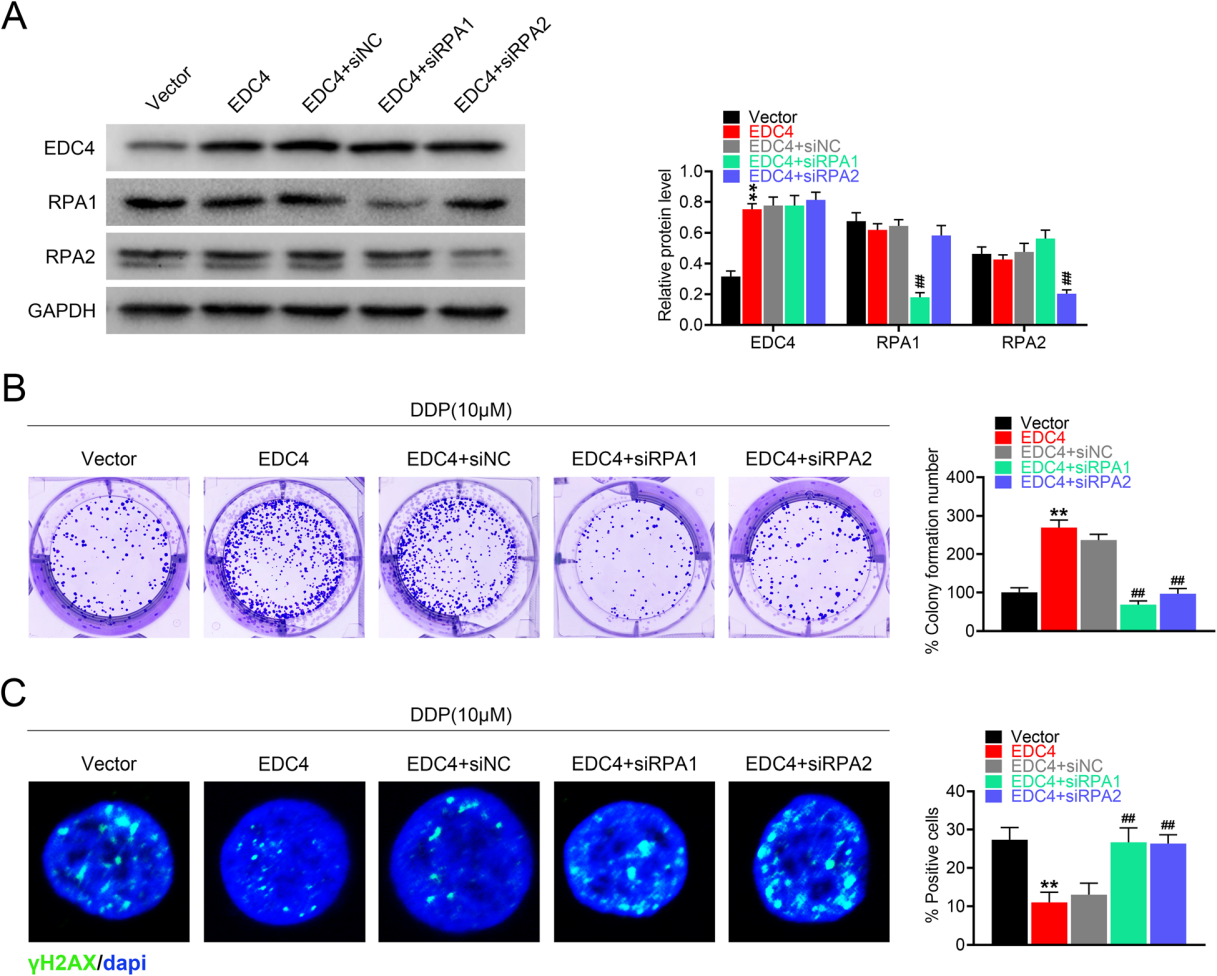
RPA knockdown reversed the inhibitory effect of EDC4 on cisplatin-induced DNA damage. **a** The level of EDC4, RPA1 and RPA2 in cells with EDC4 overexpression (or RPA knockdown) analyzed by Western blot. **b** The cell growth of HeLa (with EDC4 overexpression with/or RPA knockdown) evaluated by colony formation assay. **c** The DNA damage of HeLa (with EDC4 knockdown) analyzed by γH2AX immunofluorescent staining. **p < 0.01 vs. Vector; ^##^*p* < 0.01 vs. EDC4 + siNC

To further confirm the role of RPA on EDC4 mediated cisplatin resistance, we constructed the EDC4 over-expressed cells with RPA knockdown by siRNA transfection. The transfection efficacy was confirmed by Western blot (Fig. 5a). The level of EDC4 was increased over two times in EDC4 over-expressed cells, and RPA had no effects on EDC4 expression. The level of RPA1 or 2 decreased significantly in knockdown cells (EDC4 + siRPA1 or EDC4 + siRPA2 vs. EDC4 + siNC, *p* < 0.01). In accordance with the above results, EDC4 overexpression induced more colony formation of HeLa cells compared with negative control (EDC4 vs. Vector, *p* < 0.01), while RPA1 or 2 knockdown reversed the effects of EDC4 overexpression on colony formation (EDC4 + siRPA1 or EDC4 + siRPA2 vs. EDC4 + siNC, *p* < 0.01) (Fig. 5b). Besides, EDC4 overexpression significantly decreased the DNA damage induced by cisplatin, as the amount of γH2AX positive cells reduced in EDC4 group, which also reversed by RPA1 or 2 knockdown (Fig. 5c). These results suggested that RPA knockdown reversed the inhibitory effect of EDC4 on cisplatin-induced DNA damage.

## Discussion

Cervical cancer (CC) is the third most common gynecological malignancy around the world. Cis-Dichlorodiamineplatinum (CDDP, cisplatin) is a commonly used and one of the most effective drugs for treatment of advanced or recurrent CC [14]. However, cisplatin-resistant or drug resistance is a vital factor limiting the clinical usage of cisplatin [15]. The molecular mechanisms of cisplatin resistance are complex, and may be related with the following issues: reducing of intracellular accumulation drugs; increasing DNA repair or inactivation of apoptosis; activation of epithelial–mesenchymal transition; alteration in DNA metabolism et al [16].

Enhancer of mRNA decapping protein 4 (EDC4) is a known regulator of mRNA decapping to function within the cytoplasm and related with drug resistance. For example, researchers found that EDC4 deficiency of HeLa cells leads to hypersensitivity to DNA interstrand crosslinking drugs and PARP inhibitors, which also indicated the effects of EDC4 on DNA damage repair in the nucleus and chemo-sensitivity [9]. In this study, we examined the effect of EDC4 on cisplatin resistance in CC treatment in vitro. The present results showed that EDC4 knockdown in two cervical cancer cell lines enhanced cisplatin sensitivity by enhancing cisplatin induced cell growth inhibition and DNA damage, which was in accordance with previous report. In addition, EDC4 overexpression reduced the cisplatin induced DNA damage and enhanced cell growth of cervical cancer cells.

The mechanism of cisplatin resistance is complex. Previous reports have demonstrated that modulating cellular response to DNA replication stress is a key factor of cisplatin resistance in cancer treatment [17]. Replication protein A (RPA), a eukaryotic single-stranded DNA (ss-DNA) -binding protein, binds with high affinity to ssDNA and is responsible for regulating DNA replication, homologous recombination, nucleotide excision repair and other DNA metabolism [18, 19]. Bélanger et al. found a striking correlation between cisplatin sensitivity and the cell defects to DNA repair during S phase through RPA exhaustion, and RPA exhaustion represents a vital factor of cisplatin sensitivity [12]. Other research also demonstrated that inhibiting the activity of RPA can prevent cell cycle progression, induce cytotoxicity, and increase the efficacy of chemotherapeutic DNA-damaging agents [13]. As the present results showed that EDC4 reduced the cisplatin sensitivity in cervical cancer cells and cisplatin induced DNA damage, we speculate whether the effect of EDC4 is related to RPA. Through co-immunoprecipitation assay, the binding of EDC4 and RPA was confirmed. And further, EDC4 promoted the activation of RPA1 and RPA2. Also, RPA knockdown reversed the inhibitory effect of EDC4 on cisplatin-induced DNA damage. Liu et al. found that the C terminus of RING finger and WD repeat domain 3 (RFWD3, a substrate of checkpoint kinase ATM or ATR) could binding to RPA through the WD40 domain [20], which was also encompassed in EDC4. Thus, EDC4 may also bind to RPA through the WD40 domain. Of course, the in-depth mechanism of EDC4 and RPA in the cisplatin resistance needs further research.

## Conclusion

In conclusion, the present results indicated that EDC4 is responsible for the cisplatin resistance partly through interacting with RPA in cervical cancer by alleviating DNA damage. This study indicated that EDC4 or RPA may be novel targets to combat chemotherapy resistance in cervical cancer.

## Methods

### Cell culture

Two human cervical cancer cell lines, HeLa and SiHa, were purchased from American Type Culture Collection (ATCC, Manassas, VA, USA) and cultured in DMEM medium supplemented with 10% fetal bovine serum in a humidified incubator (37 °C, 5% CO_2_).

### Transfection

To evaluate the effect of EDC4, short hairpin RNA (shRNA) was used to establish the EDC4 knockdown cells. Human cervical cancer cell lines (HeLa or SiHa) were seeded in 6-well plates and subjected to transfection with EDC4 shRNA, or control shRNA using Lipofectamine 2000 according to the manufactory’s suggestion. To evaluate the effect of EDC4 overexpression, cells were transfected with EDC4 overexpressed plasmid or the negative control (Vector) (GenePharma, Shanghai, China) using Lipofectamine 2000 following the manufacturer’s protocol. The sequences of sh-RNAs were as follows: Sh-EDC4 #1:GGTGATAGTACCTCAGCAAAC; Sh-EDC4 #2:GCCACCCATTAACCTGCAAGA. The RPA knockdown was performed by small interfering RNA (siRNA) (synthesised by GenePharma, Shanghai, China) and transfected to cells by Lipofectamine 2000. Briefly, HeLa or SiHa cells were loaded in 6-well plate and cultured until 80% confluence. Then, the premixed lipofection and plasmids were added into the wells and incubated for 24 h. The sequences of siRNAs for RPA1 and RPA2 were referred from the previous report [21].

### MTT assay

The cell viability was analyzed by MTT assay. Briefly, cells (EDC4 knockdown) were treated with cisplatin for 48 h, and MTT (M8180-250 mg, purity ≥98%, Beijing Solarbio Science & Technology Co., Ltd., Beijing, China) solution (5 mg/mL) was added and incubated for another 4 h. DMSO was used to dissolve the formazan crystals. The absorbance at 570 nm was measured with a microplate reader (PerkinElmer Life and Analytical Sciences, Waltham, MA). The IC50 was defined as the cisplatin concentration required inhibiting cell proliferation by 50%, and calculated by SPSS.

### Colony formation assay

The transfected or cisplatin treated cells were added in plates. After the incubation of 14 days, cells were washed twice with pre-cold PBS and stained with crystal violet.. The colony formation efficiency was represented as the percentage of colonies in seeded cells number. Experiments were repeated independently in triplicate.

### Co-Immunoprecipitation (co-IP)

The immunoprecipitation was performed using Commercial immunoprecipitation kit (Sangon Biotech, Shanghai, China) according to the manufactory’s protocol. Briefly, cells of different groups were trypsinized and collected. Cells were lysed, homogenized in lysis buffer and centrifuged 12,000 rpm to collect supernatant as cell lysate. For Co-IP experiments, cell lysate was incubated with purified antibody against target protein and incubated overnight at 4 °C. Then, Protein G-conjugated beads or IgG-conjugated magnetic beads were added to lysates and incubated for 3 h. Beads were pulled down magnetically and washed with IP buffer and PBS. After elution from beads, the bounding protein was analyzed by Western Blot.

### Immunofluorescence

Cells were seeded in coverslips, fixed with 4% paraformaldehyde and permeabilized with Triton-100 (1%). After blocked with 5% goat serum, cells were incubated with primary antibody (anti-γ-H2AX, Cell Signaling, MA, USA) and then with secondary antibodies conjugated to Alexa Fluor 488. Finally, the nuclei are labeled with DAPI (4′,6-diamidino-2-phenylindole). Immunofluorescence images were captured using FV10-ASW viewer software (Olympus, Tokyo, Japan).

### Western blot

Cells were lysed in RIPA buffer containing a protease inhibitor cocktail and quantified by BCA method. Then the protein samples were added with loading buffer and heat denatured at 94 °C. The equal amount of proteins was separated using 10% SDS PAGE and transferred to PVDF membranes. Then the membranes were blocked with BSA, incubated with primary antibodies (EDC4: CST #2548; RPA1: CST #2267; RPA2: CST #35869; RPA2 (phospho T21): Abcam, ab109394) overnight at 4 °C, and finally incubated with the appropriate HRP-conjugated secondary antibodies. The protein levels were detected with ECL chemiluminescent system and the densitometry of blot was analyzed by ImageJ software.

### Real-time quantitative PCR assays

Total RNA was extracted and purified from cells by RNeasy Plus Universal Mini Kit (QIAGEN, Valencia, CA) and reverse transcribed to cDNA in a 20 μL volume. Quantitative real-time PCR analysis was performed using SYBR Green q-PCR kit (Bio-Rad, Hercules, CA). The level of the different mRNAs was normalized to GAPDH. The primer sequences refered to published paper [7].

### Statistical analysis

Statistical analysis was performed using SPSS software (SPSS Inc., Chicago, IL, USA). Data was expressed as means ± SD. Significance was analyzed using two-tailed Student’s t test. *p* value < 0.05 was considered statistically significant.

## Data Availability

All data generated or analyzed during this study are included in this published article.

## Abbreviations

CC: Cervical cancer
DAPI: 4′,6-diamidino-2-phenylindole
DDP: Cisplatin (Dichlorodiamineplatinum)
EDC4: Enhancer of mRNA decapping protein 4
RPA: Replication protein A
shRNA: short hairpin RNA
siRNA: Small interfering RNA
ss-DNA: single-stranded DNA
